# Neuropsychological and quality of life assessment in patients with Parkinson's disease submitted to bilateral deep brain stimulation in the subthalamic nucleus

**DOI:** 10.1590/S1980-57642012DN06040010

**Published:** 2012

**Authors:** Alessandra Shenandoa Heluani, Fábio Henrique de Gobbi Porto, Sergio Listik, Alexandre Walter de Campos, Alexandre Aluizio Costa Machado, Arthur Cukiert, José Oswaldo de Oliveira Jr

**Affiliations:** 1 Psychologist, Department of Psychology of Hospital “Euriclydes de Jesus Zerbini”, São Paulo SP, Brazil.; 2 MD, Neurologist. Behavioral and Cognitive Neurology Unit, Department of Neurology, and Cognitive Disorders Reference Center (CEREDIC). Hospital das Clínicas of the University of São Paulo. Department of Neurosurgery of Hospital “Euriclydes de Jesus Zerbini”, São Paulo SP, Brazil.; 3 MD, Neurosurgeon. Department of Neurosurgery of Hospital “Euriclydes de Jesus Zerbini”, São Paulo SP, Brazil. Movement Disorders Unit.; 4 MD, Neurosurgeon. Department of Neurosurgery of Hospital “Euriclydes de Jesus Zerbini”, São Paulo SP, Brazil. Movement Disorders Unit.; 5 MD, PhD. Neurologist Department of Neurology, Universidade de São Paulo, São Paulo SP, Brazil.; 6 MD, PhD. Hospital Albert Einstein, São Paulo SP, Brazil.; 7 MD, Neurosurgeon in Chief-Movement Disorders Unit - Department of Neurosurgery of Hospital “Euriclydes de Jesus Zerbini”, São Paulo SP, Brazil.

**Keywords:** deep brain stimulation, subthalamic nucleus, Parkinson's disease, cognitive assessment, quality of life

## Abstract

Deep brain stimulation (DBS) has been widely used to control motor symptoms and improve quality of life in patients with Parkinsons disease (PD). Recently, DBS in the subthalamic nucleus (STN) has become the preferred target for patients with mixed motor symptoms. Despite resultant motor and quality of life improvements, the procedure has been associated with cognitive decline, mainly in language skills, and also with psychiatric symptoms.

**Objective:**

To evaluate the influence of DBS in the STN on cognition, mood and quality of life.

**Methods:**

We studied 20 patients with PD submitted to DBS in the STN from May 2008 to June 2012 with an extensive battery of cognitive tests including memory, language, praxis, executive functions and attention assessments; the Parkinson's Disease Quality of Life Questionnaire (PDQ-39); and the Hospital Anxiety and Depression Scale (HAD), were applied both before and after the surgery. Data was analyzed using SPSS version 17.0 and results compared using the paired Student's *t* test.

**Results:**

A total of 20 patients with pre and post-operative assessments were included. A statistically significant improvement was found in total score and on subscales of mobility, activities of daily living and emotional well-being from the PDQ-39 (P=0.009, 0.025, 0.001 and 0.034, respectively). No significant difference was found on the cognitive battery or mood scale.

**Conclusion:**

DBS in the SNT improved quality of life in PD with no negative impact on cognitive skills and mood.

## Abstract (pt)

Estimulação cerebral profunda tem sido utilizada para controle das alterações motoras e melhorar qualidade de vida dos pacientes com Doença de Parkinson (DP). Mais recentemente, DBS em núcleo subtalâmico (STN) tem sido o alvo preferencialmente escolhido para sintomas mistos. Apesar da melhora motora e da qualidade de vida, o procedimento tem sido associado com declínio cognitivo, principalmente na linguagem e distúrbios psiquiátricos.

**Objetivo:**

Avaliar a influência do DBS em NST na cognição, humor e qualidade de vida.

**Métodos:**

Nós estudamos 20 pacientes submetidos a DBS em NST no período de Maio de 2008 a Junho de 2012, por meio de uma extensa avaliação neuropsicológica incluindo testes de memória, linguagem, praxia, funções executivas, funções atencionais, Parkinsons Disease Quality of Life Questionnaire (PDQ-39) e Escala Hospitalar de depressão e ansiedade (HAD) na fase pré e pós-operatória. Nós analisamos os dados usando o SPSS versão 17.0 e os resultados foram comparados através do teste pareado *t-Student*.

**Resultados:**

Houve melhora estatisticamente significativa no escore total e nas dimensões de mobilidade, atividades de vida diária e bem estar emocional do PDQ-39 (P=0,009, 0,025, 0,001 e 0,034, respectivamente). Diferenças significativas não foram encontradas na bateria cognitiva e nem na escala de humor.

**Conclusão:**

DBS em SNT melhorou a qualidade de vida nos pacientes com DP sem trazer impacto negativo nas funções cognitivas e humor.

## INTRODUCTION

Parkinson's Disease (PD) is a neurodegenerative disease that presents with motor symptoms and also with cognitive, mood, sleep and other non-motor symptoms.^1^ Besides the negative impact on quality of life caused by motor and non-motor symptoms of the disease, side effects related to treatment with medications may also occur.^2-5^ Regarding these negative aspects, surgical treatment is an option for improving motor symptoms, quality of life and the side effects of treatment with medications.

Surgical treatment for PD was first studied and applied in the United States and Europe in around 1950, during the pre Levodopa^6^ era. Technological advances in neurosciences, first with computed tomography and more recently with magnetic resonance, registry of cell activity in target nuclei and physiological stimulation with macro and micro electrodes, have improved planning, precision and surgical time.^7^ Neurosurgery is currently an important option among the PD therapeutic arsenal.

Neuromodulation is a procedure that implants deep brain electrodes (Deep Brain Stimulation-DBS), by means of stereotaxis, causing stimulation or inhibition of specific basal ganglia nuclei. DBS has been used since 1987 in the treatment of movement disorders.^8^ Currently, the anatomic targets most frequently used are the subtha-lamic nuclei (STN). DBS-STN has shown improvement in motor performance (tremor, rigidity and bradyki-nesia) in around 60% of cases and can allow the dosage levels of dopamine replacement therapy to be reduced.^9,10^

Concerning cognitive aspects in patients submitted to DBS-NST, the literature on this subject remains conflicting, perhaps due to the heterogeneity of populations and cognitive tests used in different studies. However, some studies link DBS-STN to memory, verbal fluency, executive functioning and behavioral impairments.^9,11,13,16,18^

Extensive neuropsychological assessment is crucial in the pre-operative screening of patients eligible for surgical procedures. PD is associated to several cognitive changes and the presence of dementia is a contraindication for surgery.^19,22^ Patients with PD have higher risk of developing dementia than individuals without the disease while mild cognitive impairment is a risk factor.^15,23^ It is important to evaluate patients preoperatively with a complete battery in order to gather reliable data on cognition, especially because screening tests are not sufficiently sensitive to detect post-operative changes.^21,24,25^

The selection of candidates should be multidisci-plinary and thorough given that more than 30% of postoperative problems may be due to inappropriate surgical selection.^10,14,19,20,26^ Variables to be analyzed include patient response to levodopa, presence and intensity of cognitive impairment and comorbid psychiatric illness, severity of disease and age.^10,20^

The aim of this study was to verify changes in cognition and quality of life through an extensive neuropsychological assessment in patients with PD submitted to bilateral DBS-STN.

## METHODS

Data were collected from August 2008 to June 2012, at the movement disorders unit of the Hospital "Euclydes de Jesus Zerbini". A total of 20 patients with neuropsy-chological and quality of life assessments, both pre and post-operative, were included. A semi-structured interview with patient and caregiver was then conducted to collect data on socio-demographic, clinical and functional as well as for activities of daily living. The neuropsychological assessment included the following tests: Screening: Mini-Mental State Examination (MMSE);^27^ Mood: Hospital Anxiety and Depression Scale - HADS^28^; Intellectual Quotient: Subtests Vocabulary and Matrix Reasoning (Wechsler Adult Intelligence Scale (WAIS III), when the latter could not be used to replace the block design subtest, according to the precepts of the same ar-ticle)^29^; Memory: short-term verbal memory (digit span forward) and Working memory (digit span backward)^30^; Semantic memory: Subtest Vocabulary,^30^ Episodic Verbal Memory (immediate, delayed recall and recognition) - Hopkins Verbal Learning Test-Revised - Form 1 (HVLT);^31^ Episodic Visual Memory (immediate, delayed recall and recognition) - Brief Visual Memory Test-Revised - Form 1(BVMT);^32^ Executive Functions: Verbal Fluency (FAS)^33^ and Semantic Fluency (Animals),^34^ Cognitive flexibility - Modified Wisconsin Card Sorting Test (MWCST);^35^ Processing speed - Symbol Digit Modalities Test - oral form;^36^ Language: Naming: Boston Naming Test;^37^ Attention: Selective Attention - Test of Stroop,^33^ Sustained attention and Divided - Trail Making Test A and B,^38^ Praxis: Spatial construction - Subtest Block De-sign;^30^ Functional activities: Pfeffer Functional Activities Questionnaire^39^ and Quality of Life - Parkinson's Disease Quality of Life Questionnaire (PDQ-39).^40,41^ All patients in the preoperative stage were evaluated during the "ON" state (under the influence of medication). In post-operative assessments, patients had both functioning DBS and were in use of their usual medication.

As with the PDQ-39, it was decided to collect information by interview in order to avoid interpretation errors and to optimize application time. The questionnaire comprises 39 questions divided into eight dimensions: Mobility, Activities of daily living, Emotional well-being, Stigma, Social support, Cognition, Communication and Body Discomfort.^41-43^ The total score in each dimension can vary from 0 (no problem) to 100 (maximum level of problem). Better health status is indicated by low scores and worst health status by high scores.^5,43,44^ Studies on the reliability of the Portuguese version of the instrument have been published by several different authors.^18,45,46^

Patients with serious psychiatric disorders, dementia according to current studies^11,12,17^ and those who have had bleeding or convulsion after surgical intervention were excluded.

Data analyses were done using the raw scores and percentiles of each test. Statistical analysis was done with SPSS software, version 17.0 (SPSS, Inc., Chicago, IL). The Kolmogorov-Smirnov test was used to ascertain the normality of the data, the Chi-square test for nominal variables, and the paired Student's t-test scores for comparison of pre-and post-operative data. A significance level of 0.05 was adopted for all analyses. The study was approved by the Hospital's Research Ethics Committee and all participants or their legal representatives, signed an informed consent form prior to enrolling in the study.

## RESULTS

A total of 20 patients were included (15 men and 5 women; Chi-square test [Χ^2^], p=0.025), with pre and post-operative assessments. Demographic and clinical characteristics are shown in Table 1. Mean (M) and standard deviations (SD) for the variables were: age (y) 56.8 (7.2); formal education (y) 7.5 (4.5) and time from disease onset (y) 11.5 (6), respectively. For pre-opera-tive Mini-Mental State Exam (MMSE), M and SD scores were 25.9 (2.1). The M and SD time (months) between pre and post-operative neuropsychological assessments was 9.8 (3.7); time between surgery and post-operative evaluation was 6.8 (0.7), respectively.

**Table 1:** Data on demographic and clinical characteristics.

N=20	Mean (SD)	Median	Range
Age	56.8 (7.2)	57	41-68
Education	7.5 (4.5)	5	3-17
Disease duration	11.5 (6)	11.5	1-26
MMSE	25.9 (29.4)	2.1	22-30
TPP	9.8 (3.7)	8.6	6.3-9.3
TSP	6.8 (0.7)	6.8	5.8-8.3

SD: standard seviation; TPP: time from pre to post-operative assessment; TSP: time from surgery to post-operative assessment.

Comparisons of pre and post-operative batteries are shown in Table 2. No significant difference was found in scores on cognitive batteries and mood scales, including the test of category and phonemic fluency. Table 3 shows the comparison of the PDQ-39. A statistically significant improvement was found in total score and subscales of mobility, activities of daily living and emotional well-being on the PDQ-39 (P=0.009, 0.025, 0.001 and 0.034, respectively).

**Table 2:** Comparison of pre and post-operative battery.

NPSN=20	PRE OP Mean (SD)	POST OP Mean (SD)	Diff (SD)	95% CI Diff	Sig (P two-tailed)
HAD_A - anxiety	7 (3.8)	5.5 ( 3.6)	1.5 (4.1)	[-0.4, 3.5]	0.113
HAD_D - depression	7.1 (4)	5.2 ( 2.7)	1.9 (4.1)	[-0.02, 3.8]	0.052
IQ	91.1 (12.5)	93 (12.4)	-1.8 (5.7)	[-4.5. 0.8]	0.167
Selective Attention-Stroop-colors	45.1 (21.4)	44.2 (20.2)	-1.8(12.5)	[-7.8, 4.2]	0.536
Mat _Rea	8.5 (5.2)	9.2 (6.1)	-0.7 (2.3)	[-1.8, 0.3]	0.171
SA - TMT-A	74.5 (38)	71.7 (43.2)	-3.2(33.6)	[-20.5, 13.9]	0.691
DA - TMT-B	221.4 (120.4)	211.8 (98.1)	1.8 (74)	[-36.2, 39.8]	0.068
EVMIm - HVLT	21.7 (4.3)	23.9 (6.4)	-2.2 (5)	[-4.5, 0.1]	0.841
EVM Del - HVLT	7.1 (2.2)	7.2 (2.8)	-0.1 (2.1)	[-1.1, 0.9]	0.881
EVM Rec. - HVLT	10.2 (1.5)	10.1 (1.1)	0.05 (1.4)	[-0.6, 0.7]	0.480
EViMI. - BVMT	11.6 (8.6)	12.8 (9.2)	-0.8 (5.4)	[-3.4, 1.7]	0.598
EViM Del - BVMT	4.7 (3.6)	5.2 (3.5)	-0.3 (2.5)	[-1.5, 0.9]	0.514
EViM. Rec - BVMT	4.6 (1)	5 (2.9)	-0.4 (2.7)	[-1.7, 0.9]	0.186
Short-term verbal - Digit	12.0 (3.6)	11.4 (3.2)	0.6 (1.9)	[-0.3, 1.5]	0.419
Span DO	5 (1.2)	4.8 (1.1)	0.1 (0.8)	[-0.2, 0.5]	0.171
Span IO	3.7 (1.2)	3.4 (.8)	0.2 (0.7)	[-0.1, 0.6]	0.480
Semantic Memory - Vocabulary	31.4 (9.4)	30.6 (8.4)	0.8 (4.9)	[-1.5, 3.1]	0.967
Praxis - SC	19.0 (11.6)	19.1 (12.7)	-.05 (5.3)	[-2.5, 2.4]	0.691
Cogn. Flex. - MWCST	2.7 (1.5)	2.9 (2)	-0.1 (1.6)	[-0.9, 0.6]	0.094
Verbal Fluency - FAS	29.1 (11.4)	25.6 (11.3)	3.4 (8.7)	[-0.6, 7.5]	0.081
Semantic Fluency - animals	15 (4.7)	13.3 (3.8)	1.7 (4.1)	[-0.2, 3.6]	0.406
Naming - Boston	46.1 (9.5)	46.6 (9.0)	-0.5 (2.8)	[-1.9, 0.8]	0.149
Processing speed - Symbol	29.3 (11.1)	30.8 (11.2)	-2.2 (6.3)	[-5.4, 0.8]	0.920

Cogn. Flex: cognitive flexibility; DA: divided attention - Trail Making Test B; Diff: mean difference; Post OP: post-operative assessment; Pre OP: pre-operative assessment; SD: standard deviation; DO: direct order; EviM Del: episodic visual memory delayed recall; EviM Rec: episodic visual memory recognition; EviMI: episodic visual memory immediate; EVM Del: episodic verbal memory delayed recall; EVM Del: episodic verbal memory recognition; EVM Rec. Hopkins verbal learning test-R EVMIm: episodic verbal memory immediate; HAD_A: Hospital Anxiety and Depression Scale - score anxiety; HAD_D: Hospital Anxiety and Depression Scale - score depression; HVLT: Hopkins verbal learning test-R; IO: indirect order; IQ: intellectual quotient; Mat_Rea: matrix reasoning; MWCST: Modified Wisconsin Card Sorting Test; SA: sustained attention - Trail Making Test A; Sc: spatial construction; Selective attention: stroop colors.

**Table 3:** Comparison of PDQ-39.

PDQ-39N=20	PRE OP			POST OP		Diff (SD)	Sig (P two-tailed)
	**Mean (SD)**	**Median**		**Mean (SD)**	**Median**		
Mobility	58.7 (25.1)	61.2		41.8 (21.5)	37.5	-16.8 (30.9)	0.025
Activities of daily living	59.1 (21.4)	62.5		28.4 (22.9)	24.9	-30.7 (35.6)	0.001
Emotional well-being	42.2 (22.8)	45.8		26.4 (19.6)	27	-15.8 (30.9)	0.034
Stigma	31.4 (29.4)	18.7		22.6 (24.8)	15.6	-8.8 (32.6)	0.241
Social support	10.2 (19.2)	7.6		8.8 (13.4)	0	-1.3 (23.7)	0.802
Cognition	20.2 (17.1)	15.6		20.3 (17.6)	18.7	0.1 (21.6)	0.983
Communication	33.3 (24.7)	29.1		25.8 (26.8)	16.6	-7.4 (30.3)	0.283
Body discomfort	45.8 (26)	45.8		35.3 (26.1)	37.4	-10.5 (30.7)	0.143
Total score	43.5(16.7)	43.5		28 (14.3)	23.3	-15.5(23.9)	0.009

Diff: Mean difference; Post OP: post-operative assessment; Pre OP: pre-operative assessment; SD: standard deviation. PDQ-39-subscales and total scores.

## DISCUSSION

Despite the fact that more than 70.000 patients have undergone DBS surgeries since its approval by the Food and Drug Administration (FDA) in 2002,^20^ controversies remain over the impact of DBS-NST on cognitive functions in patients with Parkinson's disease. While some studies have reported improvements in attention, executive functions and psychomotor speed, others have shown worsening in these functions as well as in memory, language, visuospatial functions and praxis. Postsurgical decline in verbal fluency has been the most consistently reported cognitive adverse effect in patients undergoing DBS-NST.^25^ Contarino et al. reviewed postoperative cognitive results at one and five years after intervention in 11 patients submitted to DBS-NST. These authors noted a worsening on fluency tests in the first postoperative year. However, three patients from the sample had preoperative cognitive impairment (two of whom had decreased verbal fluency). The latter evaluation at 5 years revealed significant decline in fluency and other cognitive domains.^47^ Zangaglia et al. evaluated PD patients submitted to surgery, comparing neuropsychological assessments at 1, 6, 12, 24 and 36 months. There were impairments in executive and verbal fluency scores in the first month, but these scores returned to pre-surgical levels after 1 year, remaining stable until the third year.^24^ In our sample, no significant cognitive change was evident upon comparing pre and post-operative neuropsychological assessments, with the latter performed between six and eight months after surgical intervention. In addition, no significant differences were observed in language and executive function tasks such as phonemic, category fluency and picture naming tests. However, scores on the phonemic fluency test (FAS) were slightly decreased after the procedure. The M and SD for pre and post-operative scores were 29.1 (11.4) and 25.6 (11.3); P=0.081, respectively.

The variation in the results reported in the literature may be due to the heterogeneity of populations for variables such as age, disease duration, concomitant medication, neuropsychological instruments, and time of follow up and reassessment. It is important to note that PD itself may be associated with cognitive decline, where an important risk factor is disease duration.^48^ Accordingly, post-operative assessment should be ideally performed 6-12 months after surgical intervention so as to avoid confounding cognitive changes due to disease progression. Significant changes shortly after the intervention are likely due to the surgical procedure and/or electrical stimulation.^25^ In our sample, average time between surgery and post-operative evaluation was 6.8 months. This is considered ideal for assessing cognitive changes reliably linked to the surgery.

The main objective of DBS is to improve quality of life and minimize motor symptoms of PD. The PDQ-39 is a well-known tool for measuring changes in quality of life.^40,41^ Erola et al. studied 29 patients at 1 and 12 months after DBS-NST with the PDQ-39. These authors found a statistically significant improvement in activities of daily living, emotional well-being, stigma and body discomfort dimensions.^49^ Nazarro et al. assessed 24 patients one-year after DBS-NST, noting a significant improvement in the domains of mobility, activities of daily living, emotional well-being, stigma, cognition and bodily discomfort.^50^ With regard to quality of life and the dimensions assessed by the PDQ-39 in the present study, significant improvement in total score and mobility, activities of daily living and emotional well-being were observed. From the patient's perspective, no changes were reported in cognitive spheres according to the analysis of "cognition" dimension of the PDQ-39. The influence of surgery on verbal fluency probably had no impact on patient's perceived cognitive function. These results are in line with those reported in the literature.^25^

DBS-STN has been associated with behavioral and psychiatric symptoms, such as apathy and increased mood, depression, suicide attempts, impulse control disorders and impulsive compulsive behaviors.^25^ The mechanism associated with these changes is not well understood. Recently, the STN has been included in the limbic and associative subcortical circuit.^25^ An anatomic gradient has been proposed, where more dorsolateral portions of the nucleus have been linked to motor functions while intermediate and anterior-medial parts seem to be associated with cognitive and emotional processes, respectively.^25^ Even with selective stimulation in dorsal portions, the energy pulse may extend to nearby ventral portions, affecting cognitive and behavioral processes. In our sample, the post-operative assessment showed a reduction in the intensity of depressive and anxiety symptoms, but these results were not statistically significant. Moreover, there was only a tendency toward reduction of depressive symptoms (P=0.052).

In conclusion, our results showed a significant improvement in the quality of life parameters of the PDQ-39 without any statistically significant cognitive impairment compared to baseline status. Functions that are usually reported to be affected by DBS-SNT, such as verbal fluency, were found to be unchanged in our sample. Despite a non-significant decrease in performance on the verbal fluency test, patients noticed improvements in quality of life and did not perceive changes in cognition (cognition dimension on PDQ-39). These findings are in line with the literature on the safety and efficacy of neurosurgery for advanced PD.^25^

The present study has some limitations. First, the sample was small, precluding the detection of small differences in cognitive scores. Second, patients were relatively young and duration of the disease long. This bias was due to the selection protocol, favoring young patients since this group benefits most from the intervention. The fact the procedure is usually indicated for moderate or advanced PD accounts for the long duration of disease. Thus, our sample is not representative of the whole population and therefore these results may not be generalized. Although a scale for depression and anxiety symptoms was employed, we did not perform screening for apathy, suicide risk, impulse control or impulsive compulsive behaviors. Finally, a learning effect (test-retest) between the two assessments cannot be ruled out, a factor which may have led to underestimation of post-operative cognitive impairment.
